# Curiosity and the desire for agency: wait, wait … don’t tell me!

**DOI:** 10.1186/s41235-021-00330-0

**Published:** 2021-11-03

**Authors:** Janet Metcalfe, Treva Kennedy-Pyers, Matti Vuorre

**Affiliations:** 1 Department of Psychology, Columbia University, New York, NY 10027 USA; 2 University of Oxford, Oxford, USA; 3 Department of Psychology, Suffolk University, Boston, USA

**Keywords:** Curiosity, Need for control, Need for agency, Active learning, Prediction error models, Reinforcement learning, Region of proximal learning, Reward learning

## Abstract

Past research has shown that when people are curious they are willing to wait to get an answer if the alternative is to not get the answer at all—a result that has been taken to mean that people valued the answers, and interpreted as supporting a reinforcement-learning (RL) view of curiosity. An alternative 'need for agency' view is forwarded that proposes that when curious, people are intrinsically motivated to actively seek the answer themselves rather than having it given to them. If answers can be freely obtained at any time, the RL view holds that, because time delay depreciates value, people will not wait to receive the answer. Because they value items that they are curious about more than those about which they are not curious they should seek the former more quickly. In contrast, the need for agency view holds that in order to take advantage of the opportunity to obtain the answer by their own efforts, when curious, people may wait. Consistent with this latter view, three experiments showed that even when the answer could be obtained at any time, people spontaneously waited longer to request the answer when they were curious. Furthermore, rather than requesting the answer itself—a response that would have maximally reduced informational uncertainty—in all three experiments, people asked for partial information in the form of hints, when curious. Such active hint seeking predicted later recall. The 'need for agency' view of curiosity, then, was supported by all three experiments.

## Introduction

It is indisputable that people both experience epistemic curiosity and that it has far reaching consequences for culture and cognition. Understanding both the conditions that promote curiosity and the mental processes associated with it is essential for teachers and learners. While some theorists view curiosity as the archetype of intrinsic motivation and active engagement (Elliot & Dweck, 2005; Ryan & Deci, 2000), others conceptualize it within reward/value-based reinforcement-learning (RL) frameworks (e.g., Berlyne, 1954, and see Friston et al., 2017; Gottlieb & Oudeyer, 2018; Gottlieb et al., 2013; Murayama et al., 2019).

The fact that people will endure adverse circumstances (Lau et al., 2020), including waiting (Marvin & Shohamy, 2016), before receiving the answers to questions about which they are curious has been offered as evidence for the RL view of curiosity, because it has been taken to indicate that people *value* (reinforcing) answers they are curious about more than those they are not curious about. The relation between waiting and value derives from research on temporal discounting investigating choices between immediate and delayed rewards. A delayed reward is chosen over an immediate reward to the extent that the agent *values* the delayed reward enough more than the immediate reward to compensate for the aversive, and devaluing (i.e., discounting) wait.

In a seminal experiment that investigated the relation between waiting and curiosity, Marvin and Shohamy (2016) presented people with trivia questions each of which was accompanied by three choices: know, wait, or skip. If participants chose either 'know' or 'skip' they were not shown the answer, but if they chose 'wait' the answer appeared after a specified amount of time. Later they were asked how curious they had been to know each answer, how satisfying each answer had been, and they were tested for recall. The finding of primary interest was that people were willing to wait to see the answers when they were curious. Memory was also better for items about which participants had been more curious, as is often observed (Bloom et al., 2018; Fastrich et al., 2018; Galli et al., 2018; Grossnickle, 2016; Gruber et al., 2014; Kang et al., 2009; Lau et al., 2020; Loewenstein, 1994; McGillivray et al., 2015; Metcalfe et al., 2017; Stare et al., 2018; Von Stumm et al., 2011; Wade & Kidd, 2019).^1^ People's willingness to wait when curious was taken to mean that they valued the items and as support for the RL view of curiosity (Marvin & Shohamy, 2016).

We do not doubt that people want to get the answer when they are curious, as was illustrated in the above experiment. Indeed, the Oxford English dictionary defines curiosity as ‘as strong desire to know.’ Nevertheless, it is possible that 'waiting' behavior was related to something other than reward/value of the answer purportedly driving the desire to know. Indeed, as noted by Loewenstein (2007), answers about which people are curious are not reliably pleasurable/rewarding and can sometimes be disappointing or silly. It is possible that something other than the 'value' of the answer may have been responsible for the waiting behavior.

We here investigate the possibility that when people are curious they have a desire to find the answer themselves. If so, they may not just be willing to wait for the answer, they may *want* to ‘wait,' if by so doing they have the opportunity to actively discover the solution themselves rather than having it given them. Many researchers have shown that people prefer situations in which they are in control (Kochanska & Aksan, 2004; Langer & Rodin, 1976). They get pleasure and self-efficacy from being in control (Bandura, 1997; Bjork & Hommer, 2007; Markant & Gureckis, 2014; Sharot et al., 2010) and will sacrifice a larger nominal reward to be in control (Bucknoff & Metcalfe, 2020). Memory is enhanced as a result of being in control (Cloutier & Macrae, 2008; Murty et al., 2015).

Furthermore, dopamine-system activation, which is associated with curiosity (Bromberg-Martin & Hikosaka, 2009; Kang et al., 2009; Lau et al., 2020), and which has been observed to be associated with primary rewards and prediction errors in RL (e.g., Bayer & Glimcher, 2005; Daw & Doya, 2006; O’Doherty et al., 2002), is also associated with agency. For instance, Leotti and Delgado (2011, 2014; Tricomi et al., 2004, and see Murayama et al., 2015) have shown enhanced dopamine/striatal activation when people make their *own agentic choices* resulting in positive outcomes as compared to equivalent rewards that occur passively. Berke (2018) has presented evidence for dopamine activation acting as an internal motivational signal ‘whether it is worth expending a limited internal resource such as energy, attention or time’ (p. 787). Berridge (2007), too, has argued that rather than being associated with either 'liking' or 'learning', dopamine is related to 'wanting' or incentive salience—a stance that is consistent with an intrinsic motivation view (Litman, 2005). Thus, although dopamine-system activation is consistent with an RL view of curiosity, it is equally consistent with a desire for agency view.

The desire to get the answer oneself is likely to be particularly strong when people feel that they have a good chance of success, as occurs when they are in their own Region of Proximal Learning (RPL, Metcalfe et al., 2021, i.e., metacognitively assessing that they are close to the answer). And, indeed, in classic RPL states, such as when people are experiencing TOTs (Brown, 1991), have made high confidence errors (Butterfield & Metcalfe, 2001), or indicate that they think they almost know the answer (Metcalfe, 2011), they show signs of being curious. In particular, they (1) allocate more study time (Son & Kornell, 2008), (2) have an augmented probability of attaining the answer spontaneously (Schwartz & Metcalfe, 2011, 2014), (3) mind wander less (Xu & Metcalfe, 2016), (4) remember better (Metcalfe & Finn, 2011; Schwartz, 1999), and (5) explicitly report being curious (Metcalfe et al., 2017).

To contrast the two hypotheses about curiosity and waiting behavior, we modified the paradigm of Marvin and Shohamy (2016). In their study, people got the answers only if they waited. In our modification, participants received the answers whether they waited or not: The timing of correct answer presentation was under participant control. If they strongly desired to get the purportedly valued answer when curious, and waiting was aversive, they could ask for the answer quickly. But if they wanted to first try to get it themselves, they might delay. We also allowed people to obtain partial information, reasoning that if people were actively trying to retrieve the answer, themselves, they might choose to take hints that confirmed or disconfirmed their conjectures—without fully unmasking the answer. If they were wrong, they could continue trying without spoiling the pleasure of actively attaining the answer themselves. Taking such hints would indicate an active stance. But if they were driven by the rewardingness of the answer itself they could simply ask for the answer at any time—reducing their uncertainty quickly and efficiently.

In summary, if the ‘desire for agency’ hypothesis was correct, people should spend more time before asking for the entire answer and should ask for hints, when they were more as compared to less curious. If the value/reward/RL hypothesis was correct, people should not bother with hints and should be quicker to request the rewarding answer the more curious they were.

## Experiment 1

### Method

Participants were 26 Columbia University undergraduates (18–27 years, 15 female, 11 male, mean age: 20.43). The number of subjects was based on previous experiments that revealed reliable within-participant curiosity differences with the same materials with group sizes of between 24 and 30 participants per condition (Bloom et al., 2018; Metcalfe et al., 2017) and considering that we planned to replicate with slight modifications. All procedures were approved by the Columbia University IRB under protocol #4902. Participants were given course credit for participation.

The materials were 128 general information questions, from the Nelson and Narens’ norms (1980) as modified by Bloom et al. (2018), that all had one-word answers (e.g., ‘What is the last name of the male star of Casablanca?’, ‘Bogart’). Participants were tested individually. Presentation order was randomized for each participant.

On each trial, participants saw a general information question and rated their curiosity on a sliding scale from ‘Not curious at all’ on the far left to ‘Very curious’ on the far right. We imposed a six-second cutoff in the curiosity-rating time, in order to isolate decision time from time 'waiting' or attempting to ascertain the answer. If they were too slow, the text ‘Too slow’ appeared with a ‘Continue’ button, which excluded the item from further consideration and skipped to the next question. As long as the participant had made the curiosity rating in time, two buttons appeared onscreen: a ‘Hint’ button and a ‘Tell me/ go on’ button. Clicking the ‘Hint’ button resulted in the computer displaying the first letter of the answer on the screen. Additional clicks revealed further letters, up to full unmasking of the target. Clicking the ‘Tell me/ go on’ button revealed the entire answer, along with a ‘Continue’ button. The question and answer remained onscreen until the participant clicked ‘Continue’ after which the next trial began.

After all 128 items had been presented, participants were tested on all questions that had not been eliminated. All choices, responses, and reaction times were recorded.

### Results

No participants were excluded from the analyses. For all analyses, we used mixed-effects regression models that specified all parameters as varying across participants and intercepts as varying across general information questions. Logistic models were used for binary outcomes, and degrees of freedom, *F* and *p* values were calculated using Satterthwaite’s method. Participants were too slow in making their curiosity ratings on 202 trials (0.06), which could not be analyzed. The data and analysis code for Experiments 1–3 are available at https://osf.io/nkqd4/.

Participants spent an average of 3.64 s (*SD* = 0.59) making initial curiosity judgments. Judgment time was uncorrelated with curiosity (mean *r* = -0.02, *p* = 0.584).

**Fig. 1:**
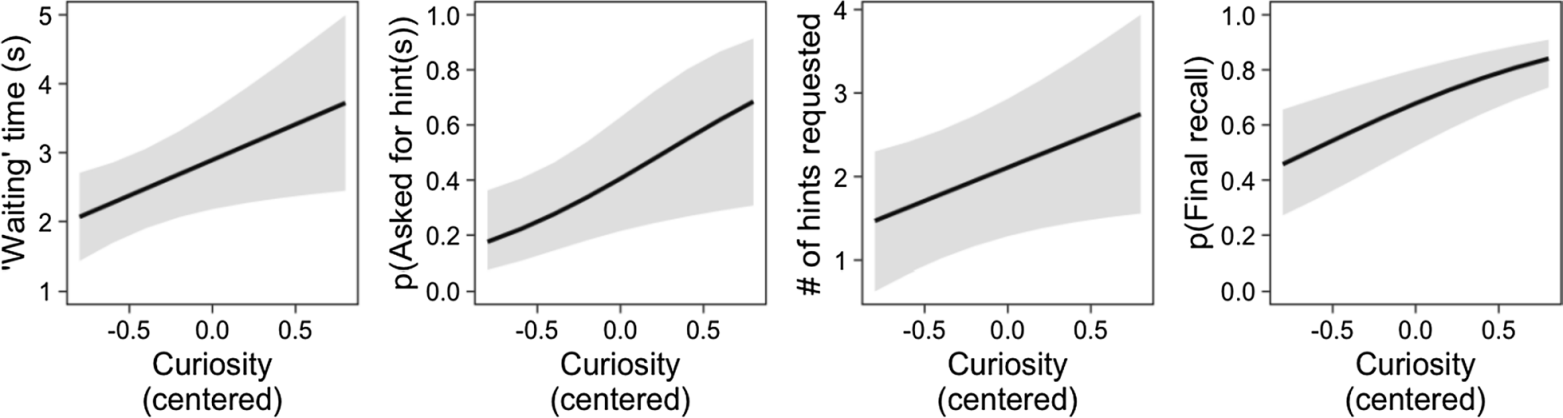
Wait times, hint seeking, and final recall as a function of curiosity, from Experiment 1. Note. Far Left: Time ‘waiting’ before requesting the answer as a function of curiosity. Left Center: Probability of asking for one or more hints as a function of curiosity. Right Center: Average number of letters (hints) viewed as a function of curiosity. Far Right: Probability of correct recall at posttest as a function of curiosity. The lines and shade represent the average relation with a 95%CI, from the mixed-effects model

*‘Waiting’ time.* The mean time spent waiting before asking for the answer—computed as the time between registering each curiosity rating and clicking the ‘Tell me/go on’ button—was 2.87 s (*SD* = 3.25). Time spent waiting increased with curiosity, as can be seen in the left panel of Fig. 1 (*b* = 1.03, *SE* = 0.45, *F*(1, 24) = 5.19, *p* = 0.032). Mean waiting times for ten curiosity-rating bins are shown in Table 1.

**Table 1:** Waiting Times by Curiosity Bin, Experiment 1

Curiosity Rating	Mean ‘wait’ time (s)	S.D. ‘wait’ time	Frequency
0.00 to 0.10	2.27	2.72	434
0.11 to 0.20	2.41	2.92	258
0.21 to 0.30	2.76	4.13	200
0.31 to 0.40	3.26	3.17	196
0.41 to 0.50	2.40	2.20	262
0.51 to 0.60	2.24	2.59	305
0.61 to 0.70	3.00	3.26	393
0.71 to 0.80	2.99	3.18	356
0.81 to 0.90	3.80	3.63	314
0.91 to 1.00	3.47	3.93	408

Mean time spent 'waiting' (i.e., the interval between making the rating of curiosity and asking for the answer to be presented), and frequency of responses, is given at each of 10 levels of curiosity which were determined by dividing the continuous curiosity scale into 10 levels. 0.0 was 'not curious at all' and 1.0 was 'extremely curious.' The starting position of the cursor on the scale was .5

*Hint seeking*. Curiosity positively predicted participants’ decision to view any hints (*b* = 1.45, *SE* = 0.61, *z* = 2.38, *p* = 0.017, left-center panel, Fig. 1). It also predicted a greater number of hints requested (*b* = 0.80, *SE* = 0.39, *F*(1, 25) = 4.17, *p* = 0.052, right-center panel).

*Time spent studying after the answer was presented.* There was no curiosity-related difference in study time once the answer was presented (mean study time = 1.52 s, *SD* = 2.23; *b* = 0.23, *SE* = 0.18, *F*(1, 10) = 1.63, *p* = 0.230). Study time is not illustrated in the figures because in none of our three experiments was there a difference in post-feedback study time as a function of pre-feedback curiosity.

*Final recall.* The far-right panel of Fig. 1 shows that curiosity positively predicted final recall (*b* = 1.15, *SE* = 0.21, *z* = 5.30, *p* < 0.001).

### Discussion

People took more time and hints before requesting the answer when they were more as compared to when they were less curious, as is consistent with the intrinsic motivation view but not with a simple RL view. Experiment 2 sought to replicate Experiment 1 with minor variations.

## Experiment 2

### Method

Twenty-four Columbia University undergraduates (18–24 years, 12 females) participated for course credit. In Experiment 2 the following changes were made: (1) Two practice trials were included, (2) curiosity ratings were made as a binary ‘Curious/Not curious’ choice, whereby participants pressed either the left (Curious) or right (Not curious) arrow keys, which were labeled on the keyboard, and (3) a ‘Continue’ button was inserted between the curiosity rating screen and the ‘Tell me/ go on’ and ‘Hint’ screen. Waiting time was still computed from the moment of making the curiosity rating until the participant pressed the 'tell me/go on' button.

Two participants were eliminated because they answered ‘Curious’ on all, or all but one, trials. Additionally, a programming error resulted in a timing error on 70 trials, leaving 2,746 observations.

### Results

Participants indicated that they were curious on 59% of the items. The time spent making the curiosity decision was 2.77 s (curious) and 2.73 s (not curious, *t*(21) = 0.75, *p* = 0.464).

**Fig. 2:**
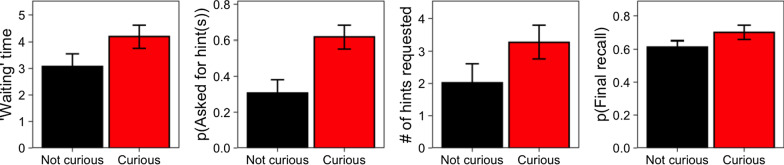
Wait times, hint seeking, and final recall by curiosity, from Experiment 2. *Note.* Far Left: Time ‘waiting’ before requesting the answer as a function of curiosity. Left Center: Probability of asking for one or more hints as a function of curiosity. Right Center: Average number of letters (hints) viewed as a function of curiosity. Far Right: Probability of correct recall at posttest as a function of curiosity. Bars and error bars represent cross-participant means with standard errors

*‘Waiting' time.* People took longer before asking for the answer when they were curious (4.19 s, *SD* = 2.00) as compared to when they were not curious (3.08 s, *SD* = 2.22, *b* = 1.08, *SE* = 0.27, *F*(1, 23) = 15.78, *p* = 0.001, Fig. 2, left panel).

*Hint seeking.* Curiosity positively predicted the decision to view hints (*b* = 2.21, *SE* = 0.45, *z* = 4.97, *p* < 0.001, Center-left panel, Fig. 2) and the number of hints that were sought: 3.27 (*SD* = 2.41) hints when curious and 2.02 (*SD* = 2.76) when not (*b* = 1.19, *SE* = 0.33, *F*(1, 21) = 12.58, *p* = 0.002, center-right panel, Fig. 2).

*Time spent studying after the answer was presented.* There was no curiosity-related difference in study time (*b* = 0.13, *SE* = 0.10, *F*(1, 114) = 1.66, *p* = 0.200).

*Final recall.* Participants correctly recalled 0.70 (*SD* = 0.20) of the items about which they had been curious and 0.61 (*SD* = 0.17) of those about which they were not curious (*b* = 0.41, *SE* = 0.21, *z* = 1.93, *p* = 0.054; Fig. 2, right panel).

## Experiment 3

An individual can lack curiosity for two reasons: they may not know the answer and not care to know it, or they may know the answer already. Unlike in the previous experiments, in Experiment 3, following Marvin & Shohamy, 2016, we excluded items about which participants claimed to already know the answer.

### Method

Twenty-four Columbia University undergraduates (18–28 years, 17 Females) participated for course credit. Experiment 3 was identical to Experiment 2, except that before the curiosity question, participants were asked if they knew the answer or not. Items that the person claimed to know were eliminated from further consideration.

### Results

A total of 1032 trials where participants reported that they knew the answer and 77 trials where participants did not provide a curiosity judgment in time were excluded. After those exclusions, two participants reported that they were ‘Curious’ on all trials and were therefore excluded. Additionally, for two participants, the first five trials were excluded because they were used as practice trials, leaving a total of 1821 trials for analyses.

Participants were curious on 63% of the questions.

**Fig. 3:**
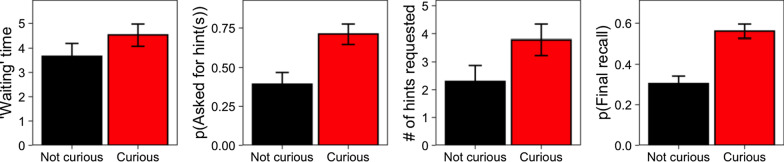
Wait times, hint seeking, and final recall by curiosity, from Experiment 3. *Note.* Far Left: Time ‘waiting’ before requesting the answer as a function of curiosity. Left Center: Probability of asking for one or more hints as a function of curiosity. Right Center: Average number of letters (hints) viewed as a function of curiosity. Far Right: Probability of correct recall at posttest as a function of curiosity. Bars and error bars represent cross-participant means with standard errors

*‘Waiting' time.* Curiosity positively predicted ‘waiting’ time (*b* = 0.84, *SE* = 0.27, *F*(1, 22) = 9.34, *p* = 0.006; Fig. 3 far-left panel).

*Hint seeking.* Curiosity positively predicted the probability of asking for hints (*b* = 2.22, *SE* = 0.43, *z* = 5.21, *p* < 0.001; Fig. 3, middle panels), and the number of hints viewed (*b* = 1.33, *SE* = 0.43, *F*(1, 17) = 9.78, *p* = 0.006).

*Time spent studying after the answer was presented.* There was no curiosity-related difference in study time (mean study time = 1.52 s, *SD* = 2.23; *b* = 0.23, *SE* = 0.18, *F*(1, 10) = 1.63, *p* = 0.230).

*Final recall.* Final recall was greater for items about which people were curious than for those about which they were not curious (*b* = 0.91, *SE* = 0.22, *z* = 4.11, *p* < 0.001; Fig. 3 far-right panel).

### Additional Analyses

Although Experiments 2 and 3 were conducted several months apart, they were otherwise identical except that in Experiment 3 the responses that people knew pre-experimentally were eliminated. The elimination of these known items produced only two significant differences: (1) Mean study time of the presented answers was shorter in Experiment 2 (1.02 s, *SD* = 0.44 s), than in Experiment 3 (1.84 s, *SD* = 1.41, *b* = 0.83, *SE* = 0.31, *F*(1, 42) = 7.03, *p* = 0.011), and (2) final recall was lower in Experiment 3 than in Experiment 2 (*b* = -1.27, *SE* = 0.39, *z* = -3.24, *p* = 0.001). Both effects were expected. All effects of curiosity, however, held up and did not differ between experiments.

We also investigated the impact on memory of active engagement as indicated by the decision to view one or more hints. Hint taking positively predicted recall in all three experiments but only reached criterial significance in Experiments 2 and 3 (Exp 1: *b* = 0.18, *z* = 1.26, *p* = 0.206; Exp 2*: b* = 0.38, *z* = 2.59, *p* = 0.01; Exp 3: *b* = 0.69, *z* = 3.17, *p* = 0.002).

**Fig. 4:**
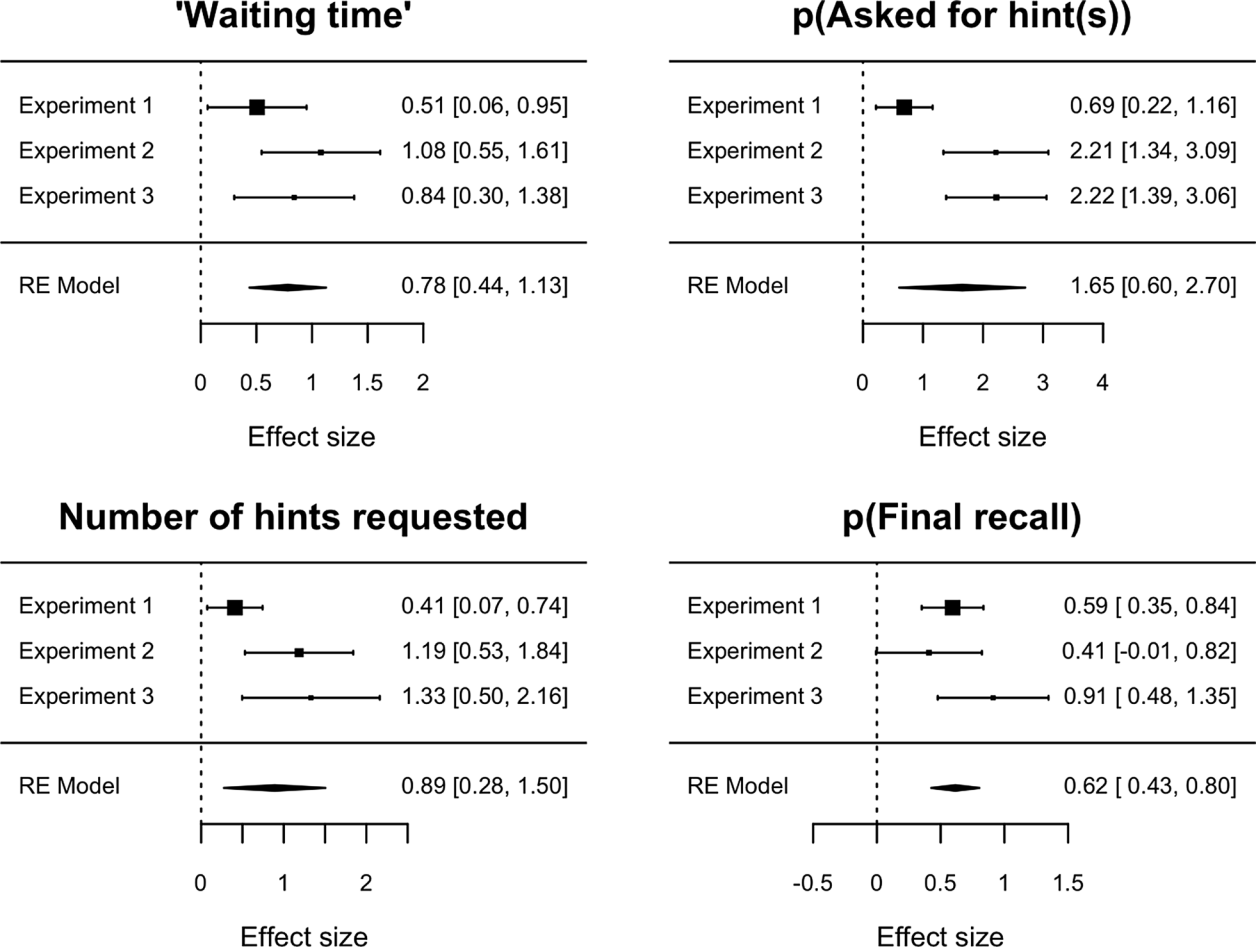
Forest plots of meta-analytic results. Note. Points and intervals indicate effects and 95%CIs (duplicated in text in the right columns). Curiosity was median-split by participant in Experiment 1 to make the estimates comparable to Experiments 2 and 3 that used binary judgments

Finally, we conducted four random-effects meta-analyses (one for each outcome) to aggregate our results (Viechtbauer, 2010). These reaffirmed the results of the individual studies (Fig. 4).

## Conclusion

Leotti et al. (2010) have argued that converging evidence from animal research, clinical studies, and neuroimaging investigations implicates the 'need for control' as a biological imperative. The data presented here suggest that this need for control, or what we have called a 'desire for agency,' is focally relevant when people are curious. People do not appear to be motivated by the reward value of the answer, per se.^2^ They did not opt to passively receive the 'reward' more quickly when they were curious, as would be the case if they were reward motivated. Rather the waiting data were consistent with the interpretation that they were trying, instead, to first get the answer themselves, especially when they were curious. A similar pattern, implicating a desire for agency, has been observed even with infants as young as 4 months old, as Sullivan and Lewis (2003) have demonstrated: A nominal informational reward was pleasurable for the infants only when it was contingent on what they, themselves, did. Consistent with the 'desire for agency' hypothesis, the three experiments presented here showed that people both delayed unveiling the answer and asked for hints, when they were curious as compared to when they were not. Finally, it was the active stance of asking for hints that predicted later memory for the answer.

### Significance statement

Scientifically understanding the conditions under which people will be curious and will both seek and learn information, as well as become more motivated to engage in further learning efforts, is a central problem for human learning in all domains, but especially in educational practice. Curiosity is often stimulated by materials that are 'on the verge' of being learned or known or are in what is called the individual's 'Region of Proximal Learning.' The present research shows that when people are in this state of curiosity, rather than wanting to merely be given the answer, they, instead, are motivated to attempt to try to get the answer by their own efforts. They choose to see hints about the answer, for example, rather than seeing the entire answer itself. Curiosity is often associated with enhanced learning. In this study it was observed that it is the propensity to ask for hints rather than the entire answer—a tendency that is itself associated with curiosity—that predicts how well the answer is learned. The desire for agency that people spontaneously displayed when they were curious in these experiments may provide clues to educators on methods to both enhance learning and to foster the pleasure people feel in learning.

## Data Availability

Data, materials, and analyses are available at https://osf.io/nkqd4/.

## Footnotes

1 It was also found that memory was improved by positive (but not negative) prediction errors, defined as satisfaction being greater than curiosity. In models like that of Rescorla and Wagner (1972) both negative and positive prediction errors—which can be thought of as surprising outcomes – are related to learning.

2 Insofar as there is something pleasurable about people finding out the answer themselves, rather than having it given to them, these results are not inconsistent with Loewenstein's (1994) 'gap' theory of curiosity although they are not predicted by it: they appear to be ‘an interesting, distinct, phenomenon’ (Loewenstein, personal communication, August 19, 2021).
